# Stroke After Influenza Vaccines in Older Adults in the US, 2016 to 2019

**DOI:** 10.1001/jamanetworkopen.2024.23926

**Published:** 2024-07-22

**Authors:** Yun Lu, Kathryn Matuska, Yuxin Ma, Layo Laniyan, Yoganand Chillarige, Steven A. Anderson, Richard A. Forshee

**Affiliations:** 1 Center for Biologics Evaluation and Research, US Food and Drug Administration, Silver Spring, Maryland; 2 Acumen LLC, Burlingame, California

## Abstract

This case series investigates whether an increase in stroke risk occurred after influenza vaccination among fee-for-service Medicare beneficiaries during the influenza seasons from 2016 to 2019.

## Introduction

The US Food and Drug Administration (FDA) investigated stroke risk following COVID-19 bivalent and influenza vaccines among Medicare beneficiaries 65 years or older in 2022 and 2023.^1^ The self-controlled case series (SCCS) study found no consistent evidence of stroke risk in the days following COVID-19 bivalent vaccination. However, an association between stroke and influenza vaccination was detected. To determine whether stroke risk increased following influenza vaccination, we investigated the 2016 to 2017, 2017 to 2018, and 2018 to 2019 influenza seasons.

## Methods

In this SCCS study, the risk of nonhemorrhagic stroke (NHS), transient ischemic attack (TIA), NHS and/or TIA (NHS/TIA), and hemorrhagic stroke (HS) following exposure to a high-dose or adjuvanted influenza vaccine was evaluated among persons 65 years or older (primary analysis) and by age subgroup (65-74, 75-84, and ≥85 years).^1,2,3^ Fixed risk windows (1-21 and 22-42 days) were compared with a control window (43-90 days). For each influenza season, the study period began on the first Sunday of August and ended 1 day before the start of the subsequent season. Participants did not reside in a nursing home and were enrolled in fee-for-service Medicare for at least 1 year before vaccination. We estimated incidence rate ratios (IRR) for each season using conditional Poisson regression and calculated the risk difference (RD) per 100 000 doses. A retrospective temporal scan identified clusters of increased stroke risk within 90 days following vaccination. Data were analyzed from August 7, 2016, to August 3, 2019. Time-fixed confounders such as race and ethnicity are implicitly controlled by the design.

This study was classified as public health surveillance and was exempted from institutional review board approval and informed consent under the Common Rule. We followed the Appropriate Use and Reporting of Uncontrolled Case Series in the Medical Literature reporting guideline.

## Results

The study population included 14 669 716 beneficiaries who received influenza vaccines across the 3 influenza seasons (median age, 74 [IQR, 69-80] years; 57.75% female). We observed 29 730 stroke cases in 2016 to 2017, 34 518 in 2017 to 2018, and 36 869 in 2018 to 2019 (Table). In 2016 to 2017, there was an association for HS during the 22- to 42-day risk window (IRR, 1.14 [95% CI, 1.02-1.28]; RD, 0.84 [95% CI, 0.14-1.54]) compared with the control interval. However, no association was identified in 2017 to 2018 or 2018 to 2019 (Figure). Temporal scans identified case clusters in control windows for several outcomes in 2017 to 2018 and 2018 to 2019.

**Table.** Observed Outcome Counts and Person-Days Among Eligible Medicare Beneficiaries Receiving High-Dose or Adjuvanted Influenza Vaccine

Influenza season	Outcome			
	NHS	TIA	NHS/TIA	HS
**2016-2017**				
No. of eligible vaccinated	8 175 412	8 175 412	8 175 412	8 175 412
Person-days	341 745 121	341 745 121	341 745 121	341 745 121
No. of cases in risk window				
1-21 d	1736	1464	3135	470
22-42 d	1708	1506	3134	549
No. of cases in control window (43-90 d)	4131	3375	7343	1144
**2017-2018**				
No. of eligible vaccinated	9 600 332	9 600 332	9 600 332	9 600 332
Person-days	401 473 253	401 473 253	401 473 253	401 473 253
No. of cases in risk window				
1-21 d	2297	1861	4060	711
22-42 d	2433	1937	4255	765
No. of cases in control window (43-90 d)	5635	4360	9782	1761
**2018-2019**				
No. of eligible vaccinated	10 499 741	10 499 741	10 499 741	10 499 741
Person-days	439 128 469	439 128 469	439 128 469	439 128 469
No. of cases in risk window				
1-21 d	2103	1700	3734	536
22-42 d	2078	1737	3722	600
No. of cases in control window (43-90 d)	4880	3974	8681	1343

Abbreviations: HS, hemorrhagic stroke; NHS, nonhemorrhagic stroke; TIA, transient ischemic attack.

**Figure.**
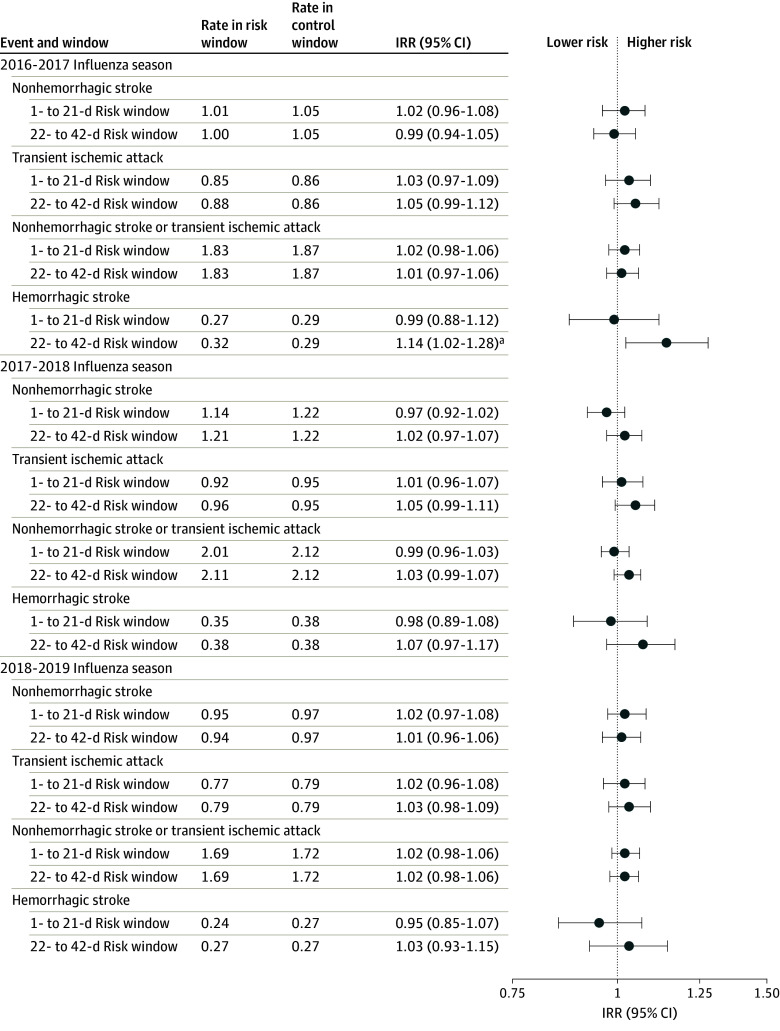
Risk of Stroke Following High-Dose or Adjuvanted Influenza Vaccines: Primary Analysis by Influenza Season Event rates in risk and control (43-90 days) windows are calculated per 100 000 person-days. Incidence rate ratios (IRRs) were calculated using conditional Poisson regression comparing the risk and control intervals. ^a^Association was present.

Subgroup analyses showed associations for HS during the 22- to 42-day risk window in those aged 65 to 74 years in 2016 to 2017 (IRR, 1.24 [95% CI, 1.02-1.51]; RD, 0.83 [95% CI, 0.07-1.59]) and 75 to 84 years in 2017 to 2018 (IRR, 1.19 [95% CI, 1.03-1.37]; RD, 1.56 [95% CI, 0.25-2.87]). In 2018 to 2019, we identified associations in the subgroup 85 years or older for NHS (IRR, 1.17 [95% CI, 1.06-1.29]; RD, 6.40 [95% CI, 2.34-10.47]) and NHS/TIA (IRR, 1.14 [95% CI, 1.06-1.23]; RD, 8.66 [95% CI, 3.51-13.81]) during the 1- to 21-day risk window.

## Discussion

Although we detected associations in the primary and age subgroup analyses, we did not observe consistent increased stroke risk following high-dose or adjuvanted influenza vaccination from 2016 to 2019. While the multiple tests conducted may have increased type I error rate, we designed our system to be sensitive and looked for consistency when interpreting results. The associations we identified were not consistent across outcomes, risk windows, age subgroups, and influenza seasons. Additionally, clusters observed in the temporal scan were not consistent with the associations identified through the SCCS framework.

Our study has some limitations. Mainly, we included only vaccinated beneficiaries. While influenza infection is a known trigger for stroke, we could not account for any protective effect of vaccination. The clinical significance of any potential risk of stroke following vaccination must be carefully considered with known benefits of influenza vaccination.^4,5^

## Notes

Supplement.
Data Sharing Statement
