# Supplementary material for: Stroke After Influenza Vaccines in Older Adults in the US, 2016 to 2019
Source: JAMA Netw Open. 2024 Jul 22;7(7):e2423926. doi: 10.1001/jamanetworkopen.2024.23926 (PMC11265121; doi:10.1001/jamanetworkopen.2024.23926)
Supplement: Supplement. — Data Sharing Statement [file jamanetwopen-e2423926-s001.pdf]

Data Sharing Statement

Lu. Stroke After Influenza Vaccines in Older Adults in the US, 2016 to 2019. *JAMA Netw Open*. Published July 22, 2024. doi:10.1001/jamanetworkopen.2024.23926

Data
Data available: No

Additional Information
Explanation for why data not available: All data produced in the present work are contained in the manuscript. Access to original datasets is not available to protect patient information.
